# Nanodiamond–Quantum Sensors Reveal Temperature Variation Associated to Hippocampal Neurons Firing

**DOI:** 10.1002/advs.202301101

**Published:** 2023-04-14

**Authors:** Giulia Petrini, Giulia Tomagra, Ettore Bernardi, Ekaterina Moreva, Paolo Traina, Andrea Marcantoni, Federico Picollo, Klaudia Kvaková, Petr Cígler, Ivo PietroDegiovanni, Valentina Carabelli, Marco Genovese*

*Adv. Sci*. **2022**, *9*, 2202014

DOI: 10.1002/advs.202202014

In the originally published article, one affiliation for P. Cígler is missing. Please find the correct affiliations below:

G. Petrini, E. Bernardi, E. Moreva, P. Traina, I. P. Degiovanni, M. Genovese

Istituto Nazionale di Ricerca Metrologica

Strada delle cacce 91, Torino 10135, Italy

E‐mail: m.genovese@inrim.it

G. Petrini, F. Picollo

Physics Department, University of Torino

via P. Giuria 1, Torino 10125, Italy

G. Petrini, G. Tomagra, A. Marcantoni, V. Carabelli

Department of Drug and Science Technology, University of Torino

Corso Raffaello 30, Torino 10125, Italy

G. Tomagra, A. Marcantoni, V. Carabelli

NIS Inter‐departmental Centre

via G. Quarello 15, Torino 10135, Italy

F. Picollo, I. P. Degiovanni, M. Genovese

Istituto Nazionale di Fisica Nucleare (INFN) Sez. Torino

via P. Giuria 1, Torino 10125, Italy

K. Kvaková, P. Cígler

Institute of Organic Chemistry and Biochemistry of the CAS

Flemingovo nam. 2

166 10 Prague 6

Czechia

K. Kvaková, P. Cígler

Institute of Medical Biochemistry and Laboratory Diagnostics

First Faculty of Medicine

Charles University

Katerinska 1660/32, Prague 2 121 08, Czechia

